# *Astragalus* and Ginseng Polysaccharides Improve Developmental, Intestinal Morphological, and Immune Functional Characters of Weaned Piglets

**DOI:** 10.3389/fphys.2019.00418

**Published:** 2019-04-12

**Authors:** C. M. Yang, Q. J. Han, K. L. Wang, Y. L. Xu, J. H. Lan, G. T. Cao

**Affiliations:** ^1^Key Laboratory of Applied Technology on Green-Eco-Healthy Animal Husbandry of Zhejiang Province, The Zhejiang Provincial Engineering Laboratory for Animal Health and Internet Technology, College of Animal Science and Technology, Zhejiang A & F University, Hangzhou, China; ^2^College of Standardization, China Jiliang University, Hangzhou, China

**Keywords:** *Astragalus* polysaccharide, ginseng polysaccharide, volatile fatty acids, microflora community, piglet

## Abstract

Antibiotic resistance is a major issue in animal industries and antibiotic-free alternatives are needed to treat infectious diseases and improve performance of pigs. Plant extracts have been suggested as a potential solution. The present study was conducted to investigate the effects of *Astragalus* polysaccharides (Aps) and ginseng polysaccharide (Gps) on growth performance, intestinal morphology, immune function, volatile fatty acids (VFAs), and microfloral community in weaned piglets. A total of 180 weaned piglets were randomly divided into three treatment groups during a 28-days feeding experiment, including a basal diet (Con), basal diet supplemented with 800 mg/kg Aps (Aps), and basal diet supplemented with 800 mg/kg Gps (Gps). Results showed that both Aps and Gps increased body weight, average daily gain and feed conversion rate, and reduced the rate of diarrhea. Gps also decreased aspartate aminotransferase compared to the Con piglets after 14 days. No significant effects on alanine aminotransferase were observed. Both Aps and Gps piglets exhibited higher serum immunoglobulin M levels after 14 and 28 days, and also decreased jejunal crypt depth, increased jejunal villus length and villus height/crypt depth ratio, and increased expression of toll-like receptor 4, myeloid differentiation primary response 88, nuclear factor-kappa B proteins in the jejunum. Aps and Gps piglets also had higher concentrations of acetic acid, isobutyric acid, and butyrate in their colon. Data of high-throughput sequencing revealed that Aps and Gps affected bacterial quantity and diversity in the colon. Species richness and evenness were higher in both Aps and Gps piglets than the control piglets. Aps and Gps piglets also had a higher relative abundance of *Lachnospiraceae* and *Anaerostipes*, and the Aps piglets had a higher relative abundance of *Lactobacillus gasseri* and *L. amylovorus*. Therefore, dietary supplementation with Aps and Gps could be beneficial for optimizing the performance of industry pigs and reducing dependence on antibiotics. Furthermore, Plant polysaccharides play a great role in promoting the sustainable development of animal husbandry.

## Introduction

Early weaning is a stressor, and sometimes exhibit intestinal development, digestive, or immune dysfunction in weaned piglets, which leads to diarrhea and growth inhibition (Hampson, 1986; Wu et al., 1996). Antibiotics are usually used to control the incidence of infectious diseases and improve growth in weaned piglets (Tang et al., 2005; Kong et al., 2007; Niekamp et al., 2007). However, antibiotic resistance has become a major issue in recent years. Hence, effective antibiotic substitutes are urgently required to reduce antibiotic dependence in the animal industry.

Plant polysaccharides have great potential as an antibiotic substitute owing to their natural characteristics (Leon et al., 2008). Reports have shown that *Astragalus* polysaccharides (Aps) extracted from *Astragalus membranaceus*, act as mild immunomodulators, restoring intestinal morphology and alleviating intestinal inflammation in acetic acid-induced colitis rats (Li et al., 2011; Zhao et al., 2014). Xu et al. (2019) reported that Aps decreased cytotoxicity and enhanced splenic lymphocyte proliferation in mice. Ginseng polysaccharides (Gps) has been shown to rescue intestinal epithelial cell viability from rotavirus infection dose-dependently (Baek et al., 2010). Kim et al. (2017) revealed that Gps exerted an anti-immunosenescent effect by suppressing thymic involution, reduced serum levels of IL-2, and modulated several types of immune cells in mice. Li et al. (2019) also showed that Gps altered the composition and diversity of gut microbiota in mice with antibiotic-associated diarrhea, restored gut microbiota, balanced metabolic processes, and promoted the recovery of mucosa.

Although the anti-inflammatory and immunomodulatory effects of plant polysaccharides have been recognized, more previous studies have focused on the immune function *in vitro* and in mice, while less on the effects of plant polysaccharides on gut volatile fatty acids (VFAs) and microbiota in weaned piglets. The present experiment was conducted to assess the effects of Aps and Gps on the growth performance, serum index, intestinal morphology, immune function, volatile fatty acid content, and colonic microbial community composition in weaned piglets. The aim was to provide valuable insights into antibiotic-free alternatives for improving weaned piglet performance.

## Materials and Methods

### Animals and Treatments

A total of 180 weaned piglets (Duroc × Landrace × Yorkshire), which weaned at day 28, were randomly assigned to three treatment groups, each group contained 6 pens per treatment, and 10 animals per pen. The experiment lasted for 29 days. The control group (Con) received a basal diet; the Aps group received the basal diet with 800 mg/kg Aps; the Gps group received the basal diet with 800 mg/kg Gps. The basal diet was formulated to meet the nutrient requirements suggested by NRC (1998) and contained no antibiotics (Table 1). Water and feed were provided *ad libitum*. All pigs were housed in a room with the temperature controlled between 26°C and 28°C. This study was carried out in accordance with the recommendations of the care and use of laboratory animals, Institutional Animal Care and Use Committee of Zhejiang Agricultural and Forestry University. The protocol was approved by the Ethics Committee of Zhejiang Agricultural and Forestry University, Hangzhou, China (SYXKzhe 2016-087).

**Table 1:** Composition and nutrient levels of the basal diet.

Ingredients	Content, %	Nutrient Level	Content
Corn	55.00	DE, MJ/Kg	14.17
Wheat midding	3.50	CP, %	20.35
Phospholipid	2.00	Lys, %	1.34
Whey powder	5.00	Met+Cys, %	0.77
Extruded soybean	7.30	Thr, %	0.80
Soybean meal	18.50	Ca, %	0.95
Fish meal	5.00	TP, %	0.65
Dicalcium phosphate	1.00	AP, %	0.48
Limestone	1.10		
NaCl	0.1		
L-Lysine HCl	0.35		
DL- methionine	0.15		
Vitamin-mineral premix^1^	1.00		
Total	100		


*^*1*^Supplied the following per kg of diet: vitamin A, 10,000 IU; vitamin D_*3*_, 400 IU; vitamin E, 10 mg; pantothenic acid, 15 mg; vitamin B_*6*_, 2 mg; biotin, 0.3 mg; folic acid, 3 mg; vitamin B_*12*_, 0.009 mg; ascorbic acid, 40 mg; Fe, 150 mg; Cu, 130 mg; Mn, 60 mg; Zn, 120 mg; I, 0.3 mg; and Se, 0.25 mg.*

### Preparation of Aps and Gps

The polysaccharide product had a purity of 80% and a molecular weight of 20,000–60,000. Aps was composed of hexanoic acid, glucose, fructose, rhamnose, arabinose, and galacturonan. Gps was composed of rhamnolipid, xylose, glucose, and galactose. Aps and Gps were provided by Vegamax Biotechnology Co., Ltd. (Anji, China).

### Growth Performance

Piglets were weighed individually at the beginning and end of experiment. Feed intake was recorded each day. Average daily feed intake (ADFI), average daily gain (ADG), and feed to gain (F:G) for each pen were calculated. The amount of diarrhea was observed daily and recorded for calculating the rate of diarrhea.

### Sample Collection

On day 14 and 28, six piglets per treatment (one piglet per pen) were randomly selected for sample collection. Blood samples were taken from the front cavity vein and centrifuged (3,000 × *g*, 10 min) at 4°C, and then the serum was separated and promptly stored at -20°C for further analysis. At 28 days, one pig per pen were slaughtered and sampled at a local commercial slaughterhouse. About 1 cm jejunal segment was immediately collected after slaughtering, and colon contents were collected and promptly stored at -80°C for the detection of VFAs and high throughput sequencing.

### Serum Parameter Analysis

Immunoglobulin A (IgA), immunoglobulin M (IgM), and immunoglobulin G (IgG) were assayed using porcine-specific immunoturbidimetry kits. Aspartate aminotransferase (AST) and alanine aminotransferase (ALT) were assayed using kits (Nanjing Jiancheng Bioengineering Institute, China), following the manufacturer’s instructions.

### Morphological and Immunohistochemical Analysis of Jejunum

The jejunum was fixed with 10% formaldehyde solution, routinely sampled, dehydrated, paraffin-embedded, sliced (4 μm thick), stained with hematoxylin-eosin, observed under an optical microscope for description (200 × ) with the main descriptive parts being photographed (Microscope: Nikon Eclipse ci; imaging system: Nikon digital sight DS-FI2). The villus height and crypt depth were measured at 10 visual fields from each intestinal sample and villus height/crypt depth ratio (VCR) values were calculated for each treatment group. Pathological changes were also observed in the same field of vision.

Immunohistochemical TLR4, MyD88, and NF-κB were performed on each sample, which antibodies were anti-TLR4 (ab8376), anti-MyD88 (ab119048) and anti-NF-kB (ab86299). Three 400-fold visual fields were randomly selected for one photography. Image-Pro Plus 6.0 software was used to select the same brown-yellow color in the blue circle as a unified criterion for judging all positive photographs. The accumulated optical density of each positive circle was obtained by analyzing the three circles of each photograph, and the average value was calculated.

### VFAs Analysis

VFA levels were tested by Headspace Sampler Gas Chromatography (Agilent Technologies, United States) using the method of Thanh et al. (2009). The commercial standards of acetic acid, butyrate, propionic acid, isovalerate, isobutyric acid, and valerate treated as external standards, were purchased from China Sinopharm Chemical Reagents Co., Ltd. (Beijing, China). Metaphosphoric acid were acquired from Shanghai Aladdin Biotech Inc. (Shanghai, China). A mixture of 1 g colon content with 6% phosphorous acid (m/v, 1:3) was injected into an Agilent Technologies GC7890 Network System (Agilent Technologies, United States) equipped with a 30 m × 0.25 mm × 0.25 μm column (HP-FFAP, Agilent Technologies) for flame ionization (Yang et al., 2018).

### Colonic Microflora Content by 16S rRNA Sequencing

Colonic content samples from each pig were used for the microbial community analysis. The V4 region of the 16S rRNA gene was detected using the Illumina-HiSeq platform (Novogene Bioinformatics Technology Co., Ltd., Beijing, China). The analysis was carried out following the method of Wang et al. (2007). The specific primer sequences 515F (5′-GTGCCAGCMGCCGCGGTAA-3′) and 806R (5′-GGACTACHVGGGTWTCTAAT-3′), were used for the analysis of V4 region of 16S rRNA gene. Briefly, through “quantitative insights into microbial ecology” (QIIME) and UPARSE software, we allocated 97% similarity between taxonomy and ribosomal database project (RDP) classifiers. Operational taxonomic units (OTUs) were clustered and species classified based on valid data. According to OTU clustering results, species annotations were made for each OTU sequence to obtain the corresponding species information and species-based abundance distribution. The alpha diversity calculations and Venn diagrams were analyzed to obtain species richness and evenness counts and information on the common and specific OTUs among different groups. Furthermore, OTUs were subjected multiple sequence alignment and a phylogenetic tree was constructed. A principal component analysis (PCA) and unweighted pair-group method with arithmetic mean (UPGMA) cluster tree displayed the differences in community structure among different groups, and results were explored in MetaStat.

### Statistical Analysis

SPSS Statistics 21.0 (SPSS Inc., United States) and GraphPad Prism 7 (GraphPad Software Inc., United States) were used for the statistical analysis. The growth performance, serum parameters, jejunal morphology, concentration of VFAs in colon contents and relative abundance of fecal microbial communities were compared and analyzed by one-way ANOVA, and results were deemed significant if *P* < 0.05.

## Results

### Effects of Aps and Gps on the Growth Performance

Piglets fed Aps or Gps had higher final body weight (*P* < 0.05) and ADG compared to the Con group (Table 2). A reduced F:G (*P* < 0.01) and diarrhea rate (*P* < 0.01) were observed in Aps and Gps piglets.

**Table 2:** Effects of the APS and GPS on growth performance of the weaned piglets^1^.

Item	Treatment			SEM^2^	*P*-value
			
	Con	Aps	Gps		
Initial BW, kg	8.72	9.06	8.98	0.11	0.47
Final BW, kg	17.56^b^	18.78^a^	18.72^a^	0.19	0.03
ADG, g	315.83^b^	347.06^a^	347.76^a^	4.68	0.01
ADFI, g	640.29^b^	618.52^c^	643.41^a^	2.53	<0.01
F: G	2.04^a^	1.84^c^	1.88^b^	0.03	<0.01
Diarrhea rate,%	6.67^a^	0.83^c^	3.33^b^	0.00	<0.01


*In the same row, values with different letter superscripts mean significant difference (*P* < 0.05). ^*1*^Con, Aps and Gps represents the piglets supplemented with basal diet, piglets supplemented with the astragalus polysaccharides and piglets supplemented with ginseng polysaccharide, respectively. ^*2*^Pooled SEM; *n* = 6 per treatment.*

### Effects of Aps and Gps on Liver Function Indexes

**FIGURE 1:**
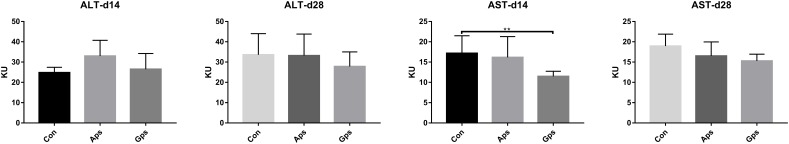
Effect of Aps and Gps on the serum liver function indexes in weaned piglets. Con represents the control piglets on d 14 and 28, respectively; Aps represents the piglets supplemented with the astragalus polysaccharide on d 14 and 28, respectively; Gps represents the piglets supplemented with ginseng polysaccharide on d 14 and 28, respectively. ^∗∗^ Means significant difference (*P* < 0.01). Values means *n* = 6 for the analysis of serum liver function indexes.

The effect of Aps or Gps on liver function indices in serum are shown in Figure 1. Gps decreased the concentration of serum AST compared to the control piglets (*P* < 0.01) at 14 days. There were no significant effects observed on the levels of ALT.

### Effects of Aps and Gps on Immunoglobulins in Serum

**FIGURE 2:**
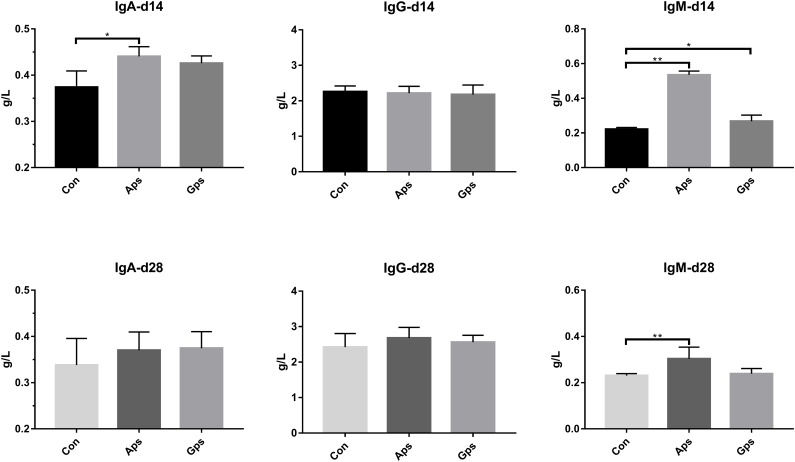
Effect of the Aps and Gps on the serum immunoglobulins in weaned piglets. Con represents the control piglets on d 14 and 28, respectively; Aps represents the piglets supplemented with the astragalus polysaccharide on d 14 and 28, respectively; Gps represents the piglets supplemented with ginseng polysaccharide on d 14 and 28, respectively. ^∗^Means significant different (*P* < 0.05), ^∗∗^Means significant difference (*P* < 0.01). Values means *n* = 6 for the analysis of serum immunoglobulins.

The effect of diet supplementation with Aps or Gps on serum immunoglobulins are shown in Figure 2. Compared to the control piglets, Aps piglets showed a higher concentration of IgA (*P* < 0.05) by 14 days, and IgM (*P* < 0.05) by 14 and 28 days. Furthermore, Gps piglets showed higher serum levels of IgM (*P* < 0.05) after 14 days.

### Effects of Aps and Gps on the Jejunal Morphology

**FIGURE 3:**
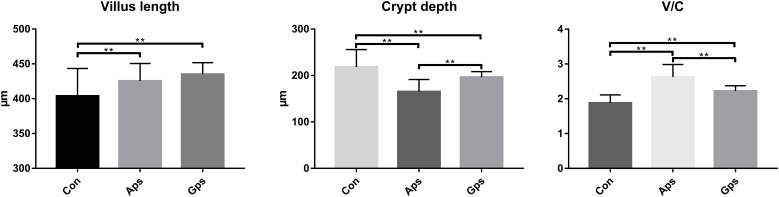
Effect of the Aps and Gps on jejunum villus of weaned piglets. A represents the villus length in the jejunum of weaned piglets. B represents the crypt depth in the jejunum of weaned piglets. C represents the ratio of the villus length and crypt depth. Con represents the control piglets; Aps represents the piglets supplemented with the astragalus polysaccharide; Gps represents the piglets supplemented with ginseng polysaccharide. ^∗∗^Means significant difference (*P* < 0.01). Values means *n* = 10 for the analysis of the jejunum form.

The effect of diet supplementation with Aps or Gps on jejunal morphology are shown in Figure 3. Compared to control group piglets, Aps or Gps decreased jejunal crypt depth (*P* < 0.01), and increased jejunal villus length (*P* < 0.01) and VCR (*P* < 0.01). In addition, there was no obvious difference regarding the jejunal villus structure between the control group and the piglets supplemented with plant polysaccharides (Supplementary Figure S1).

### Effects of Aps and Gps on Protein Expression Regarding TLR4 Signaling Pathways in Jejunal Mucosa

**FIGURE 4:**
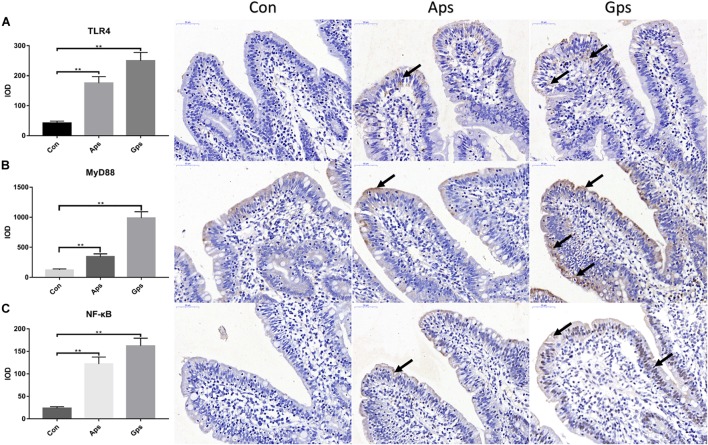
Effect of the Aps and Gps on the protein expression about TLR4 signaling pathways in jejunum of weaned piglets. A represents Immunohistochemical results of TLR4; B represents Immunohistochemical results of MyD88; C represents Immunohistochemical results of NF-κB. Con represents the control piglets; Aps represents the piglets supplemented with the astragalus polysaccharide; Gps represents the piglets supplemented with ginseng polysaccharide. Shooting multiples: 400×. ^∗∗^Means significant difference (*P* < 0.01). Values means *n* = 6 for the analysis of jejunal mucosa.

Expression of proteins involved in the TLR4 signaling pathway in the jejunal mucosa is shown in Figure 4. Relative to the control piglets, both Aps and Gps increased (*P* < 0.05) the expression of jejunal TLR4, MyD88, and NF-κB proteins. This means that the plant polysaccharides promoted the expression of proteins involved in the TLR4 signaling pathway. Furthermore, the levels of proteins involved in the TLR4 signaling pathways in the Gps piglets was higher than those in the Aps piglets.

### Effects of Aps and Gps on VFAs in Colon Contents

**FIGURE 5:**
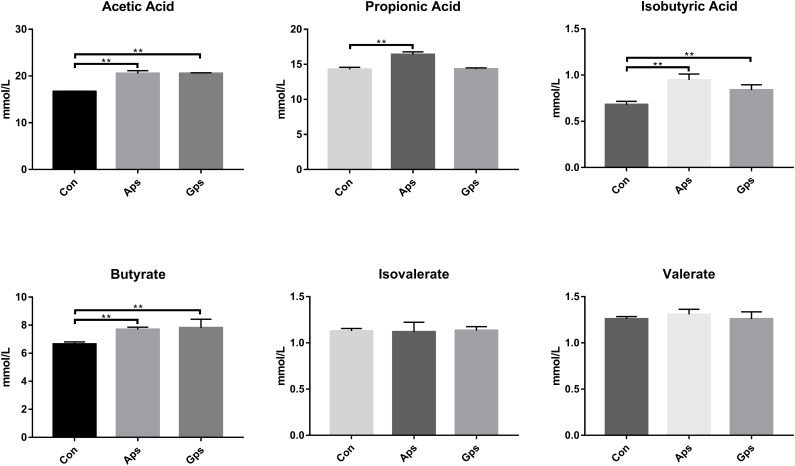
Effect of the Aps and Gps on the VFAs in colon content of weaned piglets. Con represents the control piglets; Aps represents the piglets supplemented with the astragalus polysaccharide; Gps represents the piglets supplemented with ginseng polysaccharide. ^∗∗^Means significant difference (*P* < 0.01). Values means *n* = 6 for the analysis of VFAs.

The levels of VFAs in colon contents are shown in Figure 5. The concentrations of acetic acid, isobutyric acid, and butyrate in Aps and Gps piglets were higher (*P* < 0.01) than those in the control piglets. Propionic acid was higher in Aps (*P* < 0.01) than it was in the control piglets. No significant differences in isovalerate and valerate concentrations were observed among all the different treatments.

### Effects of Aps and Gps on Colonic Microbial Community Composition

**FIGURE 6:**
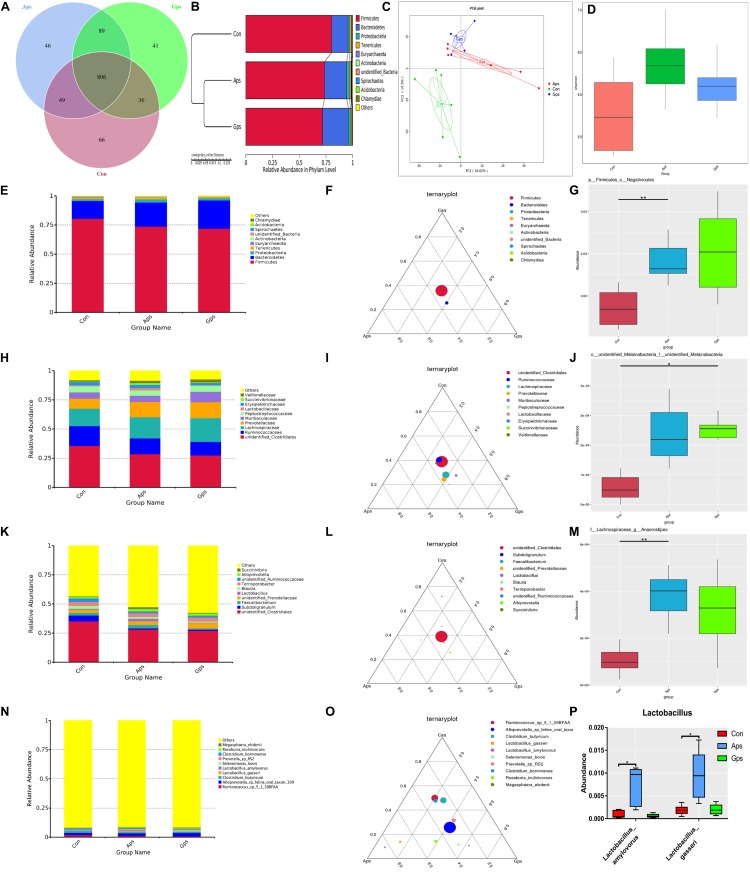
Summary of microbial community in colon contents of weaned piglets. **(A)** Represents the Venn diagram summarizing the numbers of common and unique OTUs in the microflora community in colonic contents of weaning piglets. **(B)** Represents UPGMA Cluster Tree displaying the relative abundances of predominant bacteria at the species level in each group (unweighted UniFrac distance). **(C)** Represents principle component analysis (PCA) plot about the colonic microflora. **(D)** Represents shannon index reflecting species diversity within and between groups. **(E,H,K,N)** Represent the top 10 relative abundance of microflora community (level phylum, family, genus, species). **(F,I,L,O)** Represent differences of dominant species between groups (level phylum, family, genus, species). **(G,J,M,P)** Represent a species with significant differences between groups (level phylum, family, genus, species). Con represents the control piglets; Aps represents the piglets supplemented with the astragalus polysaccharide; Gps represents the piglets supplemented with ginseng polysaccharide. OTU, operational taxonomic unit. Piglets were regarded as the experimental units, each treatment with *n* = 6. ^∗^Means different (*P* < 0.05), ^∗∗^means significant difference (*P* < 0.01).

The abundance and diversity of colonic microorganisms were described by 16S-rRNA high-throughput sequencing. The colonic microbiota composition is shown in the Venn diagram in Figure 6A. A total of 1133 OTUs were shared between the three treatment groups. The Con piglets had 66 unique OTUs, Aps piglets had 46 unique OTUs, and the Gps piglets had 41 unique OTUs. The UPGMA cluster tree and PCA both indicated that the microflora of both plant polysaccharide groups were more similar (Figure 6B,C). The alpha diversity (Shannon) was higher in both the Aps and Gps piglets than in the control group (Figure 6D).

A taxonomic classification of the microbial composition of colonic contents in piglets revealed that Firmicutes, Bacteroidetes, Proteobacteria, Tenericutes and Euryarchaeota were the dominant bacterial phyla (Figure 6E,F). The relative abundance of *Firmicutes* and *Negativicutes* in the Aps group was significantly higher (*P* < 0.05) than that in the control group (Figure 6G). At the family level, we observed that *Clostridiales*, *Ruminococcaceae*, *Lachnospiraceae*, *Prevotellaceae*, and *Muribaculaceae* were the dominant strains (Figure 6H,I). The abundance of *Melainabacteria* in the control group tended to be lower than that in the Aps and Gps groups (Figure 6J). At the genus level, the results showed that *Clostridiales*, *Subdoligranulum*, *Faecalibacterium*, *Prevotellaceae*, and *Lactobacillus* were the dominant genera in all samples (Figure 6K,L). The relative abundance of *Lachnospiraceae* and *Anaerostipes* in the Aps groups was significantly higher (*P* < 0.01) than that in control group (Figure 6M). At the species level, we found that *Ruminococcus* sp., *Alloprevotella* sp., *Clostridium butyricum*, *Lactobacillus gasseri*, and *L. amylovorus* were the dominant species in all samples. *L. gasseri* and *L. amylovorus* were higher in the Aps group (*P* < 0.01) than they were in the Con group (Figure 6N-P).

## Discussion

In recent decades, the polysaccharides from medicinal plants have attracted much attention due to their significant bioactivities, such as increasing growth performance, antioxidant activity, anti-viral activity, and immunomodulatory activities, which make them suitable as antibiotic replacements (Xie et al., 2015). Yuan et al. (2006) reported that dietary Aps increased the average daily gain of weaned piglets. The optimal Aps supplemental level was found to be between 381 mg/kg and 568 mg/kg. Han et al. (2014) reported adding 0.1% ginseng or *Acanthopanax senticosus* polysaccharides improved the growth performance and feed utilization rate in weaned piglets. Supplementation with *Atractylodes macrocephala* polysaccharides led to lower diarrheal incidence in early weaned piglets (Li et al., 2011). A study of (Li et al., 2018) showed that the supplementation of Gps modulated the intestinal microflora in rats. Current results showed that two plant polysaccharides increased growth performance, feed conversion ratio, and decreased the incidence of diarrhea in weaned piglets, especially those fed with Aps.

Plant polysaccharides have been confirmed to act as mild immunomodulators by previous studies (Yan et al., 2009; Jiao et al., 2016). Pu et al. (2015) reported that Aps decreased the activities of AST, ALT, and malondialdehyde and enhanced the activity of superoxide dismutase in carbon tetrachloride-treated mice. Liu et al. (2014) reported that 100 mg/kg of Aps not only restored the activity of serum transaminase, but also recovered intestinal pathology and ultrastructure of mice stimulated by docetaxel. Furthermore, Hu et al. (2017) reported that black ginseng significantly decreased the levels of serum ALT and AST in mice stimulated by acetaminophen. These effects were partly due to the antioxidant properties of Aps and Gps, which can scavenge free radicals, improve oxidative stress, and inhibit lipid peroxidation. Similarly, our results showed that ginseng polysaccharide reduced the levels of AST in serum on day 14.

Animal immune response to infection is closely related to immunoglobulin levels. Li et al. (2018) found that Aps alleviated a decreased serum IgG concentration in broilers treated with cyclophosphamide. Similarly, administration of Aps significantly increased the values of immune organ indices and serum IgM and IgG in mice and rats (Li et al., 2009; Meng et al., 2017). Tan et al. (2017) found that dietary supplementation with Aps significantly enhanced the presence of IgG and IgM in sow blood. Lan et al. (2016) indicated that pigs fed with *A. membranaceus* had higher IgG and IgA concentrations than those fed the control diet. The Aps injection increased the content of IgG, IgM, and IgA in weaned piglets (Xie et al., 2015). In addition, research found that the Gps increased the number of NK cells in the blood of immunosuppressed mice and promoted the expression of perforin and granzyme (Sun et al., 2016). We found that adding Aps could increase IgA and IgM levels in piglets after 14 and 28 days compared with control piglets. The results indicated that Aps and Gps could enhance immune function in piglets.

Wang et al. (2015) evaluated the immunomodulating activities of Aps in chicks. Aps was found to increase jejunal villus height at 8 mg/kg body weight. In rat, Aps in the range of 50–200 μg/ml significantly increased the number of intestinal epithelial cells (IEC-6), remarkably increased cell migration rate at the wound site and accelerated the recovery of tissue calluses (Zhang et al., 2014). Lv et al. (2017) indicated that the addition of 200 μg/ml Aps significantly increased the density of intestinal cells and the regular distribution of intestinal microvilli by increasing the transcription and expression of cytokeratin (CK18, ZO-2, etc.). Cui et al. (2018) found that *A. membranaceus* decoction restored villus height and V/C of duodenum, jejunum, and ileum under lipopolysaccharides stimulation in mice. We found that both dietary Aps and Gps enhanced the intestinal healthy development of weaned piglets by decreasing crypt depth and increasing villus height and intestinal gland ratio in weaned piglets.

TLR4 to nuclear factor-kappa B (TLR4-NF-κB) pathway is involved in pathogen identification and infection defense in mammals (Zhang et al., 2017). Aps enhanced immune response including anti-tumor activities (Li et al., 2018). Wang et al. (2017) reported that Aps-mediated immunomodulatory activities in macrophages are involved in TLR4/NF-κB signaling pathways. Zhou et al. (2017) found that Aps stimulated the key nodes in the TLR4-MyD88-dependent signaling pathway, including TLR4, MyD88, TRAF-6, and NF-κB, both *in vitro* and in mice. Wang et al. (2018) confirmed that the NF-κB mRNA and protein levels were significantly increased in macrophages treated with Aps. These findings were similar to ours, in that both Aps and Gps increased expression of jejunal TLR4, MyD88, and NF-κB proteins, which means that the plant polysaccharides promoted the expression of proteins involved in the TLR4 signaling pathway. Wei et al. (2016) revealed that Aps enhanced the production of the tumor necrosis factor (TNF-α) that is blocked by the inhibitor of TLR4. The above results demonstrated that plant polysaccharides may regulate host immune functioning by activating the TLR4-mediated MyD88-dependent signaling pathway.

Generally, cecal and colonic VFAs were indicators of intestinal health and microbial activity (Bindelle et al., 2009). The cecal and colonic microorganisms ferment polysaccharides to produce VFAs such as acetic acid, propionic acid, and butyric acid. Che et al. (2018) indicated that supplementation with *A. membranaceus* significantly increased intestinal acetic acid, isobutyric acid, butyrate, and total VFAs in piglets. While, no trials have been conducted to investigate the effects of Gps on the colonic VFAs in weaned piglets. In the present study, piglets supplemented with Aps or Gps tended to have markedly higher concentrations of acetic acid, isobutyric acid, and butyrate.

The colon is the main habitat for microorganisms that affect host growth, development, and health (Pajarillo et al., 2014; Kim et al., 2015). Che et al. (2018) established that supplementation with 2.5–5% of *A. membranaceus* fiber decreased intestinal pH and increased intestinal levels of VFAs and gut microbial population and diversity, which enhances the health and well-being of piglets. In our case, Firmicutes and Bacteroidetes were the advantage bacterial phyla. Lamendella et al. (2011) pointed out that *Firmicutes* and *Bacteroides* were the dominant bacteria in pig feces, which was supported by our data. Moreover, supplementation with Aps and Gps significantly increased the levels of *Firmicutes* and *Negativicutes*, which may improve unsaturated fatty acid synthesis in geese (Chen et al., 2018). Furthermore, Aps and Gps increased the relative abundance of Melainabacteria and *Selenomonas* in the colon. Also, Melainabacteria was found to be beneficial to the host because it fermented plant fibers in the gut and synthesized vitamin B and vitamin K (Di et al., 2013; Soo et al., 2014).

At the genus level, we observed that the levels of *Lanchnospiraceae* and *Anaerostipes* in the Aps piglets were significantly higher than those in the control group. *Anaerostipes* was shown to increase the level of acetic acid, propionic, and isobutyric acid in human feces (Bier et al., 2018). At the species level, our sequencing results indicated that *L. gasseri* and *L. amylovorus* were the dominant bacterial species in Aps piglets. *Lactobacillus* are recognized probiotics. Oh et al. (2016, 2018) reported that *L. gasseri* showed greater inhibition of nitric oxide production, considerably higher antioxidant activity and inhibition of α-glucosidase activity than other strains, which led to improved immune health of cows by fermenting lactic acid, propionic acid, and modulating pro-inflammatory cytokines. Similar findings were reported by Nobutani et al. (2017), who explained that *L. gasseri* significantly improved intestinal irritable bowel syndrome severity. *L. amylovorus* has also been found to modulate the negative regulators Tollip, interleukin-1 receptor-associated kinase, and extracellular heat shock proteins (Hsp72 and Hsp90) to achieve anti-inflammatory effects (Finamore et al., 2014). Furthermore more, Martinez et al. (2013) reported that *L. amylovorus* promoted short chain fatty acids and ammonia in feces of humans and piglets. In the present study, piglets supplemented with Aps or Gps enhanced the concentration of VFAs by increasing the microbial population and diversity in the colon of piglets. The present results suggest that growth performance was increased by modulating the microbial populations and increasing colonic microflora, which may be relative to the improvement of VFAs and intestinal morphology.

## Conclusion

In conclusion, dietary supplementation with Aps or Gps could improve the growth performance, liver function, and intestinal villus morphology, and may regulate host immune functioning by activating the TLR4-mediated MyD88-dependent signaling pathway, and also enhance the concentration of VFAs in the colon and increase the colonic microbial population and diversity in weaned piglets. Plant polysaccharides may regulate the final fermentation products and the number and diversity of bacteria in the colon to promote the healthy growth of piglets.

## Data Availability

The raw data supporting the conclusions of this manuscript will be made available by the authors, without undue reservation, to any qualified researcher.

## Ethics Statement

This study was carried out in accordance with the recommendations of the care and use of laboratory animals, Institutional Animal Care and Use Committee of Zhejiang Agricultural and Forestry University. The protocol was approved by the Ethics Committee of Zhejiang Agricultural and Forestry University, Hangzhou, China (SYXKzhe 2016-087).

## Author Contributions

CY, QH, and GC designed the trials, performed the experiments, and edited the manuscript. KW and YX performed the samples detection. YX, KW, and JL analyzed the data. QH wrote the manuscript, which was edited by CY and GC. All authors read and approved the final manuscript.

## Conflict of Interest Statement

The authors declare that the research was conducted in the absence of any commercial or financial relationships that could be construed as a potential conflict of interest.

## Supplementary Material

Figure S1 Effect of Aps and Gps on the morphology of jejunum villus in weaned piglets. Con represents the control piglets; Aps represents the piglets supplemented with the astragalus polysaccharide; Gps represents the piglets supplemented with ginseng polysaccharide. Shooting multiples: 200×.

## References

[B1] Baek S.-H. Lee J. G. Park S. Y. Bae O. N. Kim D.-H. Park J. H. (2010). Pectic polysaccharides from panax ginseng as the antirotavirus principals in ginseng. *Biomacromolecules* 11 2044–2052. 10.1021/bm100397p 20597500

[B2] Bier A. Braun T. Khasbab R. Di Segni A. Grossman E. Haberman Y. (2018). A high salt diet modulates the gut microbiota and short chain fatty acids production in a salt-sensitive hypertension rat model. *Nutrients* 10:1154. 10.3390/nu10091154 30142973 PMC6164908

[B3] Bindelle J. Buldgen A. Delacollette M. Wavreille J. Agneessens R. Destain J. P. (2009). Influence of source and concentrations of dietary fiber on in vivo nitrogen excretion pathways in pigs as reflected by in vitro fermentation and nitrogen incorporation by fecal bacteria123. *J. Anim. Sci.* 87 583–593. 10.2527/jas.2007-0717 18791157

[B4] Che D. Adams S. Wei C. Gui-Xin Q. Atiba E. M. Hailong J. (2018). Effects of Astragalus *membranaceus* fiber on growth performance, nutrient digestibility, microbial composition, VFA production, gut pH, and immunity of weaned pigs. *MicrobiologyOpen.* 10.1002/mbo3.712 [Epub ahead of print]. 30117299 PMC6528644

[B5] Chen X. Liu X. Du Y. Wang B. Zhao N. Geng Z. (2018). Green forage and fattening duration differentially modulate cecal microbiome of Wanxi white geese. *PLoS One* 13:e0204210. 10.1371/journal.pone.0204210 30252869 PMC6155509

[B6] Cui Y. Wang Q. Sun R. Guo L. Wang M. Jia J. (2018). Astragalus *membranaceus* (Fisch.) Bunge repairs intestinal mucosal injury induced by LPS in mice. *BMC Compl. Altern. Med.* 18:230. 10.1186/s12906-018-2298-2 30075775 PMC6091064

[B7] Di R. C. Sharon I. Wrighton K. C. Koren O. Hug L. A. Thomas B. C. (2013). The human gut and groundwater harbor non-photosynthetic bacteria belonging to a new candidate phylum sibling to Cyanobacteria. *eLife* 2:e01102. 10.7554/eLife.01102 24137540 PMC3787301

[B8] Finamore A. Roselli M. Imbinto A. Seeboth J. Oswald I. P. Mengheri E. (2014). *Lactobacillus amylovorus* inhibits the TLR4 inflammatory signaling triggered by enterotoxigenic *Escherichia coli* via modulation of the negative regulators and involvement of TLR2 in intestinal Caco-2 cells and pig explants. *PLoS One* 9:e94891. 10.1371/journal.pone.0094891 24733511 PMC3986366

[B9] Hampson D. J. (1986). Alterations in piglet small intestinal structure at weaning. *Res. Vet. Sci.* 40:32 10.1016/S0034-5288(18)30482-X 3704321

[B10] Han J. Bian L. Liu X. Zhang F. Zhang Y. Yu N. (2014). Effects of *Acanthopanax senticosus* polysaccharide supplementation on growth performance, immunity, blood parameters and expression of pro-inflammatory cytokines genes in challenged weaned piglets. *Asian-Australas. J. Anim. Sci.* 27 1035–1043. 10.5713/ajas.2013.13659 25050047 PMC4093559

[B11] Hu J. N. Liu Z. Wang Z. Li X. D. Zhang L. X. Li W. (2017). ameliorative effects and possible molecular mechanism of action of black ginseng (*Panax ginseng*) on acetaminophen-mediated liver injury. *Molecules* 22:664. 10.3390/molecules22040664 28430162 PMC6154718

[B12] Jiao R. Liu Y. Gao H. Xiao J. So K. F. (2016). The anti-oxidant and antitumor properties of plant polysaccharides. *Am. J. Chin. Med.* 44 463–488. 10.1142/s0192415x16500269 27109156

[B13] Kim J. Nguyen S. G. Guevarra R. B. Lee I. Unno T. (2015). Analysis of swine fecal microbiota at various growth stages. *Arch. Microbiol.* 197 753–759. 10.1007/s00203-015-1108-1 25832348

[B14] Kim M. Yi Y.-S. Kim J. Han S. Y. Kim S. H. Seo D. B. (2017). Effect of polysaccharides from a Korean ginseng berry on the immunosenescence of aged mice. *J. Ginseng Res.* 42 447–454. 10.1016/j.jgr.2017.04.014 30337804 PMC6187098

[B15] Kong X. Yin Y. Guoyao W. U. Liu H. Yin F. Tiejun L. I. (2007). Dietary supplementation with *Acanthopanax senticosus* extract modulates cellular and humoral immunity in weaned piglets. *Asian Aust. J. Anim. Sci.* 20 1453–1461. 10.5713/ajas.2007.1453

[B16] Lamendella R. Santo Domingo J. W. Ghosh S. Martinson J. Oerther D. B. (2011). Comparative fecal metagenomics unveils unique functional capacity of the swine gut. *BMC Microbiol.* 11:103. 10.1186/1471-2180-11-103 21575148 PMC3123192

[B17] Lan R. X. Park J. W. Lee D. W. Kim I. H. (2016). Effects of Astragalus *membranaceus*, *Codonopsis pilosula* and allicin mixture on growth performance, nutrient digestibility, faecal microbial shedding, immune response and meat quality in finishing pigs. *J. Anim. Physiol. Anim. Nutr.* 101 1122–1129. 10.1111/jpn.12625 27868250

[B18] Leon C. G. Tory R. Jia J. Sivak O. Wasan K. M. (2008). Discovery and development of toll-like receptor 4 (TLR4) antagonists: a new paradigm for treating sepsis and other diseases. *Pharm. Res.* 25 1751–1761. 10.1007/s11095-008-9571-x 18493843 PMC2469272

[B19] Li J. Li R. Li N. Zheng F. Dai Y. Ge Y. (2018). Mechanism of antidiabetic and synergistic effects of ginseng polysaccharide and ginsenoside rb1 on diabetic rat model. *J. Pharm. Biomed. Anal.* 158 451–460. 10.1016/j.jpba.2018.06.024 30032757

[B22] Li J. Zhong Y. Li H. Zhang N. Ma W. Cheng G. (2011). Enhancement of Astragalus polysaccharide on the immune responses in pigs inoculated with foot-and-mouth disease virus vaccine. *Int. J. Biol. Macromol.* 49 362–368. 10.1016/j.ijbiomac.2011.05.015 21640133

[B24] Li R. Chen W. C. Wang W. P. Tian W. Y. Zhang X. G. (2009). Extraction, characterization of Astragalus polysaccharides and its immune modulating activities in rats with gastric cancer. *Carbohydr. Polym.* 78 738–742. 10.1016/j.carbpol.2009.06.005

[B25] Li S. Qi Y. Chen L. Qu D. Li Z. Gao K. (2019). Effects of Panax ginseng polysaccharides on the gut microbiota in mice with antibiotic-associated diarrhea. *Int. J. Biol. Macromol.* 124 931–937. 10.1016/j.ijbiomac.2018.11.271 30503788

[B26] Liu W. Gao F. F. Li Q. Lv J. W. Wang Y. Hu P. C. (2014). Protective effect of Astragalus polysaccharides on liver injury induced by several different chemotherapeutics in mice. *Asian Pac. J. Cancer Prev.* 15 10413–10420. 10.7314/APJCP.2014.15.23.10413 25556485

[B27] Lv J. Zhang Y. Tian Z. Liu F. Shi Y. Liu Y. (2017). Astragalus polysaccharides protect against dextran sulfate sodium-induced colitis by inhibiting NF-κÂ activation. *Int. J. Biol. Macromol.* 98 723–729. 10.1016/j.ijbiomac.2017.02.024 28188801

[B28] Martinez R. C. Cardarelli H. R. Borst W. Albrecht S. Schols H. Gutiérrez O. P. (2013). Effect of galactooligosaccharides andBifidobacterium animalisBb-12 on growth of *Lactobacillus amylovorus* DSM 16698, microbial community structure, and metabolite production in anin vitrocolonic model set up with human or pig microbiota. *FEMS Microbiol. Ecol.* 84 110–123. 10.1111/1574-6941.12041 23167835

[B29] Meng F. X. Xu P. J. Wang X. Huang Y. Wu L. Y. Chen Y. L. (2017). Investigation on the immunomodulatory activities of extracts in a cyclophosphamide (CTX)-induced immunosuppressanted mouse model. *Saudi Pharm. J.* 25 460–463. 10.1016/j.jsps.2017.04.006 28579875 PMC5447429

[B30] Niekamp S. R. Sutherland M. A. Dahl G. E. Salak-Johnson J. L. (2007). Immune responses of piglets to weaning stress: impacts of photoperiod1. *J. Anim. Sci.* 85 93–100. 10.2527/jas.2006-153 17179544

[B31] Nobutani K. Sawada D. Fujiwara S. Kuwano Y. Nishida K. Nakayama J. (2017). The effects of administration of the *Lactobacillus gasseristrain* CP2305 on quality of life, clinical symptoms and changes in gene expression in patients with irritable bowel syndrome. *J. Appl. Microbiol.* 122 212–224. 10.1111/jam.13329 27761980

[B32] Oh N. S. Joung J. Y. Lee J. Y. Kim Y. (2018). Probiotic and anti-inflammatory potential of *Lactobacillus rhamnosus* 4B15 and *Lactobacillus gasseri* 4M13 isolated from infant feces. *PLoS One* 13:e0192021. 10.1371/journal.pone.0192021 29444150 PMC5812581

[B33] Oh N. S. Lee J. Y. Kim Y. (2016). The growth kinetics and metabolic and antioxidant activities of the functional synbiotic combination of *Lactobacillus gasseri* 505 and *Cudrania tricuspidata* leaf extract. *Appl. Microbiol. Biotechnol.* 100 10095–10106. 10.1007/s00253-016-7863-3 27796437

[B34] Pajarillo E. A. B. Chae J. P. Balolong M. P. Kim H. B. Kang D. K. (2014). Assessment of fecal bacterial diversity among healthy piglets during the weaning transition. *J. Gen. Appl. Microbiol.* 60 140–146. 10.2323/jgam.60.140 25273987

[B35] Pu X. Fan W. Yu S. Li Y. Ma X. Liu L. (2015). Polysaccharides from *Angelica* and Astragalus exert hepatoprotective effects against carbon-tetrachloride-induced intoxication in mice. *Can. J. Physiol. Pharmacol.* 93 39–43. 10.1139/cjpp-2014-0331 25415237

[B36] Soo R. M. Skennerton C. T. Sekiguchi Y. Imelfort M. Paech S. J. Dennis P. G. (2014). An expanded genomic representation of the Phylum *Cyanobacteria*. *Genome Biol. Evol.* 6 1031–1045. 10.1093/gbe/evu073 24709563 PMC4040986

[B37] Sun Y. Guo M. Feng Y. Zheng H. Lei P. Ma X. (2016). Effect of ginseng polysaccharides on nk cell cytotoxicity in immunosuppressed mice. *Exp. Therapeu. Med.* 12 3773–3777. 10.3892/etm.2016.3840 28105109 PMC5228343

[B38] Tan L. Wei T. Yuan A. He J. Liu J. Xu D. (2017). Dietary supplementation of Astragalus polysaccharides enhanced immune components and growth factors EGF and IGF-1 in sow colostrum. *J. Immunol. Res.* 2017:9253208. 10.1155/2017/9253208 28164139 PMC5253170

[B39] Tang Z. R. Yin Y. L. Nyachoti C. M. Huang R. L. Li T. J. Yang C. (2005). Effect of dietary supplementation of chitosan and galacto-mannan-oligosaccharide on serum parameters and the insulin-like growth factor-I mRNA expression in early-weaned piglets. *Domest. Anim. Endocrinol.* 28 430–441. 10.1016/j.domaniend.2005.02.003 15826777

[B40] Thanh N. T. Loh T. C. Foo H. L. Hair-bejo M. Azhar B. K. (2009). Effects of feeding metabolite combinations produced by *Lactobacillus plantarumon* growth performance, faecal microbial population, small intestine villus height and faecal volatile fatty acids in broilers. *Br. Poult. Sci.* 50 298–306. 10.1080/00071660902873947 19637029

[B41] Wang D. D. Pan W. J. Mehmood S. Chen X. D. Chen Y. (2018). Polysaccharide isolated from *Sarcodon aspratus* induces RAW264.7 activity via TLR4-mediated NF-κB and MAPK signaling pathways. *Int. J. Biol. Macromol.* 120(Pt A), 1039–1047. 10.1016/j.ijbiomac.2018.08.147 30171950

[B42] Wang Q. Garrity G. M. Tiedje J. M. Cole J. R. (2007). Naive bayesian classifier for rapid assignment of rrna sequences into the new bacterial taxonomy. *Appl. Environ. Microbiol.* 73 5261–5267. 10.1128/aem.00062-07 17586664 PMC1950982

[B43] Wang X. Li Y. Shen J. Wang S. Yao J. Yang X. (2015). Effect of Astragalus polysaccharide and its sulfated derivative on growth performance and immune condition of lipopolysaccharide-treated broilers. *Int. J. Biol. Macromol.* 76 188–194. 10.1016/j.ijbiomac.2015.02.040 25748840

[B44] Wang Z. Liu Z. Zhou L. Long T. Zhou X. Bao Y. (2017). Immunomodulatory effect of APS and PSP is mediated by Ca2-cAMP and TLR4/NF-κB signaling pathway in macrophage. *Int. J. Biol. Macromol.* 94 283–289. 10.1016/j.ijbiomac.2016.10.018 27732877

[B45] Wei W. Xiao H. T. Bao W. R. Ma D. L. Leung C. H. Han X. Q. (2016). TLR-4 may mediate signaling pathways of Astragalus polysaccharide RAP induced cytokine expression of RAW264.7 *cells*. *J. Ethnopharmacol.* 179 243–252. 10.1016/j.jep.2015.12.060 26743224

[B46] Wu G. Meier S. A. Knabe D. A. (1996). Dietary glutamine supplementation prevents jejunal atrophy in weaned pigs. *J. Nutr.* 126 2578–2584. 10.1093/jn/126.10.2578 8857520

[B47] Xie J. H. Jin M. L. Morris G. A. Zha X. Q. Chen H. Q. Yi Y. (2015). Advances on bioactive polysaccharides from medicinal plants. *Crit. Rev. Food Sci. Nutr* 56 S60–S84. 10.1080/10408398.2015.1069255 26463231

[B48] Xu S. Wusiman A. Liu Z. Gu P. Ni H. Zhang Y. (2019). pH-responsive Astragalus polysaccharides-loaded poly(lactic-co-glycolic acid) nanoparticles and their in vitro immunogenicity. *Int. J. Biol. Macromol.* 125 865–875. 10.1016/j.ijbiomac.2018.12.156 30576729

[B49] Yan F. Zhang Q. Y. Jiao L. Han T. Zhang H. Qin L. P. (2009). Synergistic hepatoprotective effect of *Schisandrae lignans* with Astragalus polysaccharides on chronic liver injury in rats. *Phytomedicine* 16 805–813. 10.1016/j.phymed.2009.02.004 19345075

[B50] Yang C. M. Zhang L. L. Cao G. T. Feng J. Yue M. Xu Y. L. (2018). Effects of dietary supplementation with essential oils and organic acids on the growth performance, immune system, faecal volatile fatty acids and microflora community in weaned piglets. *J. Anim. Sci.* 97 133–143. 10.1093/jas/sky426 30388227 PMC6312551

[B51] Yuan S. L. Piao X. S. Li D. F. Kim S. W. Guo P. F. (2006). Effects of dietary astragalus polysaccharide on growth performance and immune function in weaned pigs. *Animalence* 82 501–507. 10.1079/ASC200653

[B52] Zhang C. L. Ren H. J. Liu M. M. Li X. G. Sun D. L. Li N. (2014). Modulation of Intestinal epithelial cell proliferation, migration, and differentiation in vitro by Astragalus Polysaccharides. *PLoS One* 9:e106674. 10.1371/journal.pone.0106674 25157577 PMC4144960

[B53] Zhang R. Yu Q. Shi G. Liu R. Zhang W. Zhao X. (2017). chTLR4 pathway activation by Astragalus polysaccharide in bursa of fabricius. *BMC Vet. Res.* 13:119. 10.1186/s12917-017-1039-y 28464901 PMC5414374

[B54] Zhao L. Wu H. Zhao A. Lu H. Sun W. Ma C. (2014). The in vivo and in vitro study of polysaccharides from a two-herb formula on ulcerative colitis and potential mechanism of action. *J. Ethnopharmacol.* 153 151–159. 10.1016/j.jep.2014.02.008 24548752

[B55] Zhou L. Liu Z. Wang Z. Yu S. Long T. Zhou X. (2017). Astragalus polysaccharides exerts immunomodulatory effects via TLR4-mediated MyD88-dependent signaling pathway in vitro and in vivo. *Sci. Rep.* 7:44822. 10.1038/srep44822 28303957 PMC5355992

