# Supplementary figures and images for: Astragalus and Ginseng Polysaccharides Improve Developmental, Intestinal Morphological, and Immune Functional Characters of Weaned Piglets
Source: Front Physiol. 2019 Apr 12;10:418. doi: 10.3389/fphys.2019.00418 (PMC6473041; doi:10.3389/fphys.2019.00418)

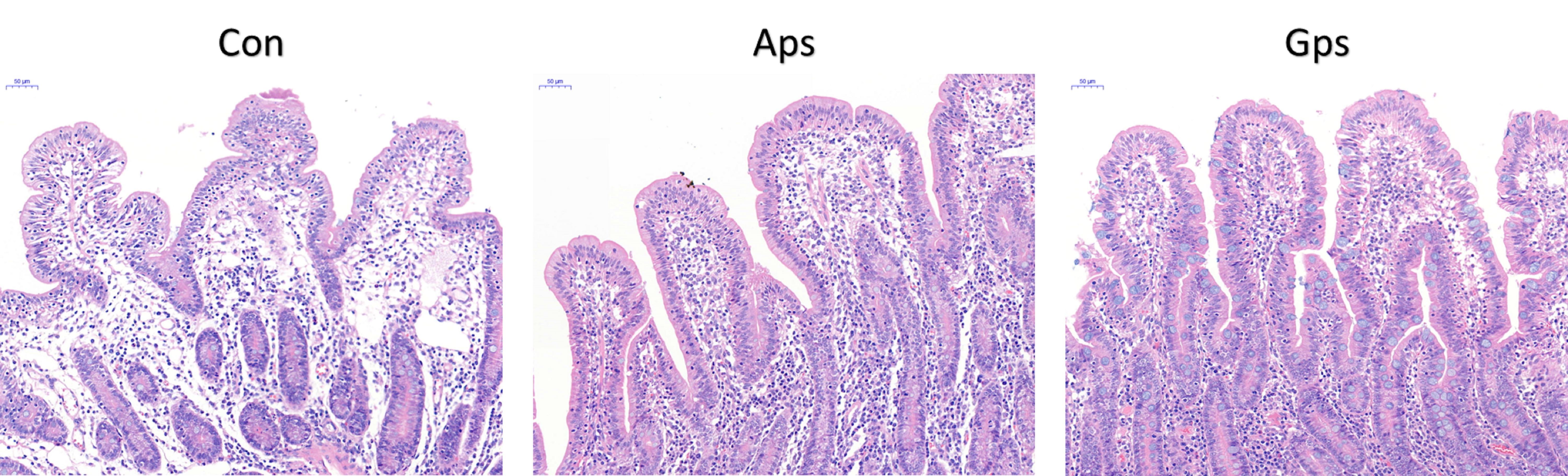

Supplement: Figure S1 — Effect of Aps and Gps on the morphology of jejunum villus in weaned piglets. Con represents the control piglets; Aps represents the piglets supplemented with the astragalus polysaccharide; Gps represents the piglets supplemented with ginseng polysaccharide. Shooting multiples: 200×. [file Image_1.TIF]
